# Transaortic Catheter Venting for Left Ventricular Unloading in Veno-Arterial Extracorporeal Life Support: A Porcine Cardiogenic Shock Model

**DOI:** 10.3390/medicina61040552

**Published:** 2025-03-21

**Authors:** Sang Yoon Kim, Hyoung Woo Chang, Jae Hang Lee, Jae Hyun Jeon, Yoohwa Hwang, Hwan Hee Park, Dong Jung Kim

**Affiliations:** 1 Department of Thoracic and Cardiovascular Surgery, Seoul National University Bundang Hospital, Seongnam-si 13620, Gyeonggi-do, Republic of Korea; sangyun87@gmail.com (S.Y.K.); chang.hyoungwoo@gmail.com (H.W.C.); truemed@hotmail.com (J.H.L.); fine1114@hanmail.net (J.H.J.); ghksgl12@gmail.com (H.H.P.); 2 Heartwell Vein Clinic, Seoul 04783, Republic of Korea; syooflower@gmail.com; 3 Department of Thoracic and Cardiovascular Surgery, Bucheon Sejong Hospital, Bucheon-si 14754, Gyeonggi-do, Republic of Korea

**Keywords:** porcine model, hypoxic biventricular dysfunction, transaortic catheter venting, veno-arterial extracorporeal membrane oxygenation

## Abstract

*Background and Objectives*: Left ventricle (LV) overloading during veno-arterial (VA) extracorporeal membrane oxygenation (ECMO) is detrimental to myocardial recovery. To determine whether LV unloading using transaortic catheter venting (TACV) is effective, we analyzed the effect of TACV in a human-sized porcine model. *Materials and Methods*: Hypoxic biventricular dysfunction was induced in 11 pigs using femoro-femoral VA-ECMO and custom-made TACV catheters in the LV through the common carotid artery. Hemodynamic conditions were then simulated. The TACV was either opened or closed under a controlled ECMO flow. Conversely, the ECMO flow was adjusted, varying from 1 L to 4 L, with and without TACV; 2115 observations were collected. *Results*: In comparing observations without TACV (TACV−) and with TACV (TACV+), the change in left ventricular end-diastolic pressure (LVEDP) after TACV application was −1.2 mmHg (*p* < 0.001). In the linear regression model, the reduction in LVEDP was maximized when the baseline LVEDP and ECMO flow were higher. When escalating the ECMO flow in the respective settings of TACV− and TACV+, the rise in LVEDP was significantly lower in TACV+. *Conclusions*: TACV decreased LVEDP; this effect was more prominent when ECMO flow and baseline LVEDP were higher. These findings suggest that TACV might support LV recovery through effective unloading, even when ECMO flow is high.

## 1. Introduction

The role of veno-arterial (VA) extracorporeal membrane oxygenation (ECMO) in the treatment of cardiogenic shock has been established [1,2,3]. However, left ventricle (LV) distension during VA-ECMO, which causes pulmonary edema, LV thrombosis, and myocardial injury, is a major concern when LV dysfunction is so severe that the opening of the aortic valve is limited [4]. However, VA-ECMO alone has limitations in the salvage of critical patients, and even early initiation of VA-ECMO in cardiogenic shock failed to improve mortality and necessitated additional mechanical circulatory support in a recent randomized controlled trial [5]. Especially when the signs of LV dysfunctions, including low pulse pressures in the arterial line, pulmonary edema with foamy secretion, and infrequent opening of the aortic valve in echocardiography are combined, LV unloading using another mechanical circulatory support is required [6]. In this situation, direct LV decompression using a catheter-delivered microaxial pump (Impella; ABIOMED Inc., Danvers, MA, USA) is a good adjunct to ECMO [7,8,9,10]. However, in many developing countries, the industrial support and medical costs of this device make it difficult to apply this technology in practice. Besides medical costs and availability, there are some limitations with and concerns about the microaxial pump, including risks of acute brain injury including stroke, which makes it contraindicated when the LV thrombus is observed [11,12]. A recent meta-analysis presented that current LV unloading strategies still have complications and prolonged recovery times, although they have shown decreased mortality compared to no-unloading strategies [13].

As an alternative method of percutaneous direct LV unloading, transaortic catheter venting (TACV) of the LV using a pigtail catheter has been introduced in small case series and preclinical studies [14,15,16]. While Kitamura et al. [16] showed echocardiographic unloading of LV by TACV in a porcine model, Jung et al. [15] presented the feasibility of TACV in humans and its effectiveness in hemodynamic improvement. However, current evidence is limited due to the small number of sample sizes in the preclinical studies and clinical studies demonstrating retrospective data of non-comparative single-arm cohorts. Questions regarding how much flow can be achieved through TACV, under what condition TACV is needed and effective, whether it is enough for the unloading of the LV, and how the hemodynamic change by peripheral VA-ECMO differs between patients with and without TACV have not yet been answered in clinical or preclinical studies [17]. These questions necessitate comprehensive, quantitative, and statistical analyses of the hemodynamic effects of TACV. This study aimed to analyze the effects of TACV on peripheral VA-ECMO in a human-sized porcine model.

## 2. Materials and Methods

### 2.1. Study Design and Subjects

This study was supported by Grant No. 02-2021-0010 from the Seoul National University Bundang Hospital Research Fund. This study was approved by the Seoul National University Bundang Hospital Institutional Animal Care and Use Committee (SNUBH-IACUC, BA-2109-327-001-08), and all experiments and procedures were conducted ethically in conformity with animal rights. Regarding the execution, analysis, and reporting of the experiment, the authors adhered to Animal Research: Reporting of In Vivo Experiments (ARRIVE) guidelines 2.0.

Between November 2021 and February 2023, 17 pigs were used in this study. Excluding the first six pigs, which were lost during ECMO cannulation and catheterization, 2115 observations from 11 pigs were acquired. The baseline characteristics of the animals are summarized in Table A1.

### 2.2. Procedures and Protocols

Pigs were intravenously anesthetized with zoletil (5 mg/kg) and xylazine HCl (2.2–4.4 mg/kg), followed by endotracheal intubation and inhalation anesthesia using sevoflurane. Under general anesthesia, both the common femoral arteries and veins were cannulated with 7 Fr. vascular sheaths, and one of the two femoral arterial sheaths was connected to a pressure monitoring kit. Blood gas analysis and electrolyte assays were also performed. Subsequently, both common carotid arteries were cannulated with 7 Fr. vascular sheaths. The right internal jugular vein was cannulated with a 9 Fr. Arrowg + ard Blue^®^ MAC catheter (Teleflex, Morrisville, NC, USA). Following the loss of experimental subjects due to vessel injury during cannulation, all the aforementioned cannulation procedures were performed using a 5 Fr. Micropuncture Access Set (COOK MEDICAL LLC, Bloomington, IN, USA) under ultrasound guidance without surgical incision.

**Figure 1:**
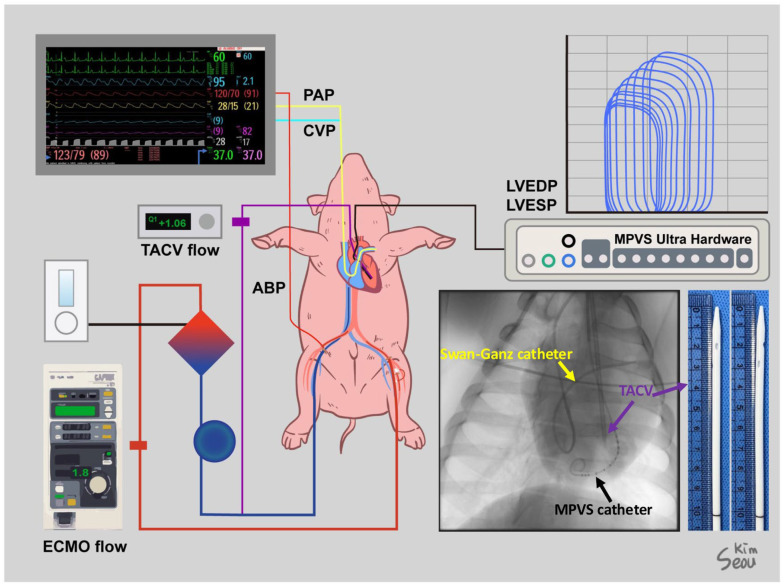
Settings for the experiment of a human-sized acute hypoxic biventricular dysfunction porcine model under extracorporeal membrane oxygenation (ECMO) and transaortic catheter venting (TACV). Sites of cannulation and lines for monitoring were described, and a simple X-ray shows a Swan–Ganz catheter, Millar Pressure-Volume System (MPVS) catheter, and TACV catheter. A photo of the TACV catheter was taken.

After a heparin (25,000 IU) injection, a Swan–Ganz catheter was inserted through the MAC catheter under fluoroscopic guidance to monitor pulmonary arterial pressure (PAP) and cardiac output (CO). A VentriCath 507 catheter (Millar Inc., Houston, TX, USA) was inserted through one of the common carotid arterial sheaths into the LV supported by a 7 Fr. ENVOY guiding catheter (Integra Life Sciences, Princeton, NJ, USA) under fluoroscopic guidance and connected to a Millar Pressure-Volume System (Millar, Inc., Houston, TX, USA). The location of the Ventri-Cath 507 catheter tip was modified according to the optimal position to consistently monitor the pressure and volume. Initial hemodynamic data, including chamber pressures and initial CO, were collected. ECMO cannulation was performed through the femoral artery and vein sheath with a 15 Fr. arterial and 17 Fr. venous Biomedicus cannulas (Medtronic Inc., Minneapolis, MN, USA). For TACV, an inner introducer of a 17 Fr. venous cannula with an outer diameter of 12 Fr. was modified by creating side holes and inserted through a common carotid arterial sheath over a Safari wire (Boston Scientific Inc., Marlborough, MA, USA), which was adequately firm to guide and support the passage of the TACV into the LV. Fluoroscopy confirmed that the tip of the TACV was located in the LV outflow tract. The transaortic catheter was directly connected to a 1/4″ PVC line and extended to a 3/8″ PVC line using a 1/4–3/8″ connector to monitor the isolated flow of the TACV using a NovaFlow ultrasonic flow meter (Novalung GmbH, Heilbronn, Germany). The aforementioned cannulas and monitoring lines are graphically summarized in Figure 1.

To induce hypoxic biventricular dysfunction, the ECMO sweep gas flow and ventilator were repetitively turned off for 10–15 min until the systolic arterial blood pressure (ABP) dropped below 60 mmHg and was restarted. After five to six hypoxic injuries, the CO was measured using a Swan–Ganz catheter and confirmed to be reduced to less than 50% of the initial value. The aforementioned procedure for inducing hypoxic biventricular dysfunction was repeated because myocardial function was restored during ECMO support and the TACV.

**Figure 2:**
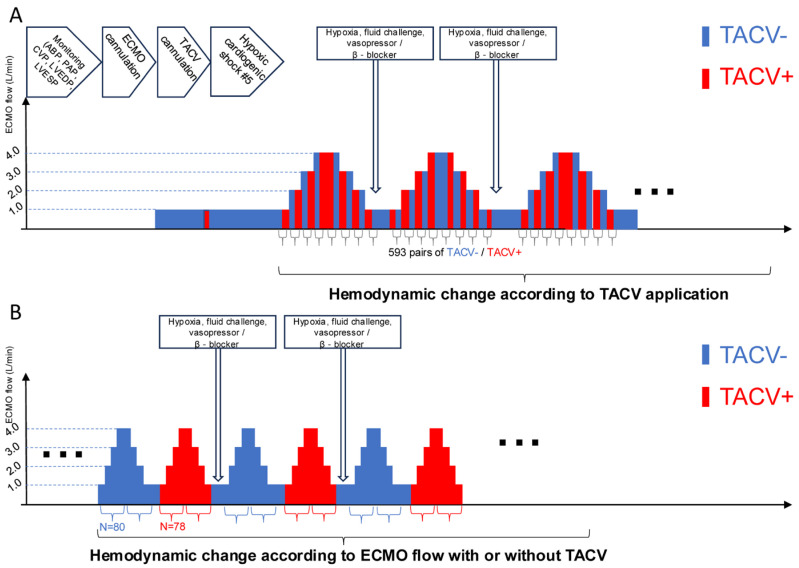
Procedure sequence and protocols of data acquisition. (**A**) After initial induction of acute hypoxic biventricular dysfunction, hemodynamic parameters were measured while the TACV was turned on and off in every extracorporeal membrane oxygenation (ECMO) flow. (**B**) Hemodynamic parameters were measured while ECMO flow was changed up and down between 1 L/min and 4 L/min with TACV (TACV−) or without TACV (+). ABP, arterial blood pressure; CVP, central venous pressure; LVEDP, left ventricular end-diastolic pressure; LVESP, left ventricular end-systolic pressure; MPVS, Millar Pressure-Volume System; PAP, pulmonary artery pressure.

Multiple hemodynamic situations were simulated by volume challenge with a 0.5–2 L infusion of normal saline, afterload adjustment with norepinephrine (0.1–1.0 µg/kg/min), and suppression of contractility with labetalol infusion (10–40 µg/kg/min). The TACV was opened or closed under fixed ECMO flow (Figure 2A). Conversely, the ECMO flow changed from 1 L to 4 L with or without TACV (Figure 2B). Hemodynamic data, including ABP, PAP, central venous pressure (CVP), LV end-diastolic pressure (LVEDP), LV end-systolic pressure (LVESP, defined as the trough LV pressure before diastolic filling), and heart rate, were collected repeatedly when hypoxic injury, ECMO flow, TACV, volume challenge, and intravenous medicine changes occurred.

### 2.3. Statistical Analysis

Continuous variables were summarized as mean ± standard deviation when normally distributed or when the number of measurements was over 30, according to the central limit theorem. The significance of their change or difference was tested using the Student’s *t*-test and paired *t*-test. The other small number of continuous variables were described as the median and interquartile range (25th to 75th percentile), and their changes or differences were tested using the Mann–Whitney U test.

The effects of TACV and ECMO flow were analyzed based on paired observations of individual subjects. The observations for the groups with and without TACV and ECMO flow from 1 L to 4 L were compared, followed by correlation analysis using the Pearson correlation coefficient and linear regression analysis for LVEDP change according to TACV and ECMO flow change. All statistical tests were performed using IBM SPSS Statistics for Windows, Ver. 26.0 (IBM Corp. Armonk, NY, USA), and a *p*-value of 0.05 or less was considered statistically significant.

## 3. Results

### 3.1. Hemodynamic Change According to TACV Application

Based on the ECMO rotations per minute, number of hypoxic injuries, volume challenge, and norepinephrine and labetalol infusion rates, 593 pairs of observations with and without TACV were used for the analysis (Figure 2A). The changes in hemodynamic measurements in the TACV group (TACV+) compared with those in the no-TACV group (TACV−) are summarized in Table 1. While the TACV flow was approximately 15% of the total ECMO flow, the total ECMO flow increased (+175.4 mL, *p* < 0.001), although drainage from the systemic venous drain decreased in the TACV+ (−256.5 mL, *p* < 0.001). Although the change in mean ABP was not significant (*p* = 0.058), pulse pressure was lower in TACV− (−1.8 mmHg, *p* < 0.001). As a consequence, systolic ABP was higher in TACV− (1.5 mmHg, *p* < 0.001), and diastolic ABP was higher in TACV + (0.4 mmHg, *p* = 0.032). The LVEDP and LVESP were significantly lower in the TACV + LVEDP (−1.2 mmHg, *p* < 0.001 LVESP. −1.1 mmHg, *p* < 0.001).

**Figure 3:**
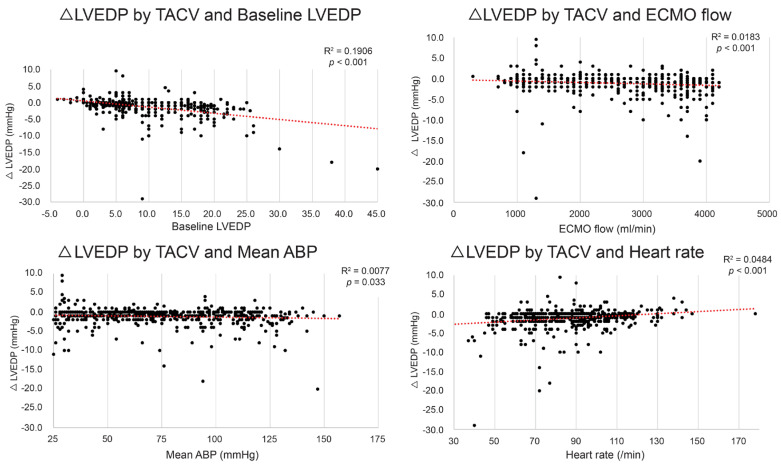
Change in left ventricle end-diastolic pressure (ΔLVEDP) by transaortic catheter venting (TACV) application according to baseline LVEDP, extracorporeal membrane oxygenation (ECMO) flow, mean arterial blood pressure (ABP), and heart rate. The decrease in LVEDP was more prominent when baseline LVEDP, ECMO flow, and mean ABP were higher, and heart rate was lower. The red dashed lines represent the linear trend lines.

Although the mean LVEDP difference between TACV− and TACV+ was −1.2 mmHg, the difference varied depending on the baseline hemodynamic conditions, with reductions in LVEDP exceeding 5 mmHg observed in certain settings. To determine the conditions in which the hemodynamic circumstance LVEDP decrease by TACV is maximized, the LVEDP change between TACV− and TACV+ (ΔLVEDP; LVEDP in TACV+ –LVEDP in TACV−) was analyzed in relation to baseline hemodynamic variables. Baseline LVEDP, ECMO flow, mean ABP, and CVP were significant independent predictive factors of decreased LVEDP after TACV (Table 2 and Figure 3).

While high LVEDP was defined as baseline LVEDP > 20 mmHg, a favorable response was defined as ΔLVEDP ≤ −5 mmHg. Favorable responses in the high LVEDP group were grouped into the effective TACV group, and trials that failed to induce favorable responses were grouped into the ineffective TACV group. Hemodynamic measurements were compared between the two groups (Table 3). When applied in a high-baseline LVEDP setting, TACV was more effective when ECMO flow was higher, pulse pressure was lower, and heart rate was slower.

### 3.2. Hemodynamic Change According to ECMO Flow with or Without TACV

**Figure 4:**
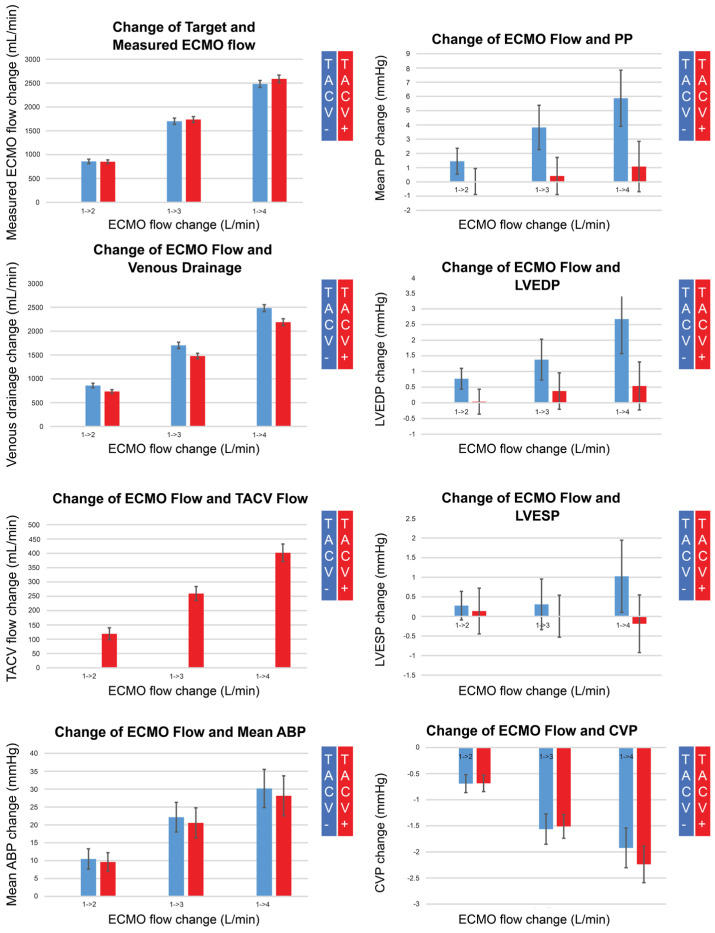
Changes in measured extracorporeal membrane oxygenation (ECMO) flow, venous drainage, transaortic catheter venting (TACV) flow, mean arterial blood pressure (ABP), pulse pressure (PP), left ventricular end-diastolic pressure (LVEDP), left ventricular end-systolic pressure (LVESP), and central venous pressure (CVP), according to changes in ECMO flow without TACV (TACV−) or with TACV (TACV+). In TACV+, the increase in LVEDP according to elevated ECMO flow was significantly lower than that in TACV−.

**Figure A1:**
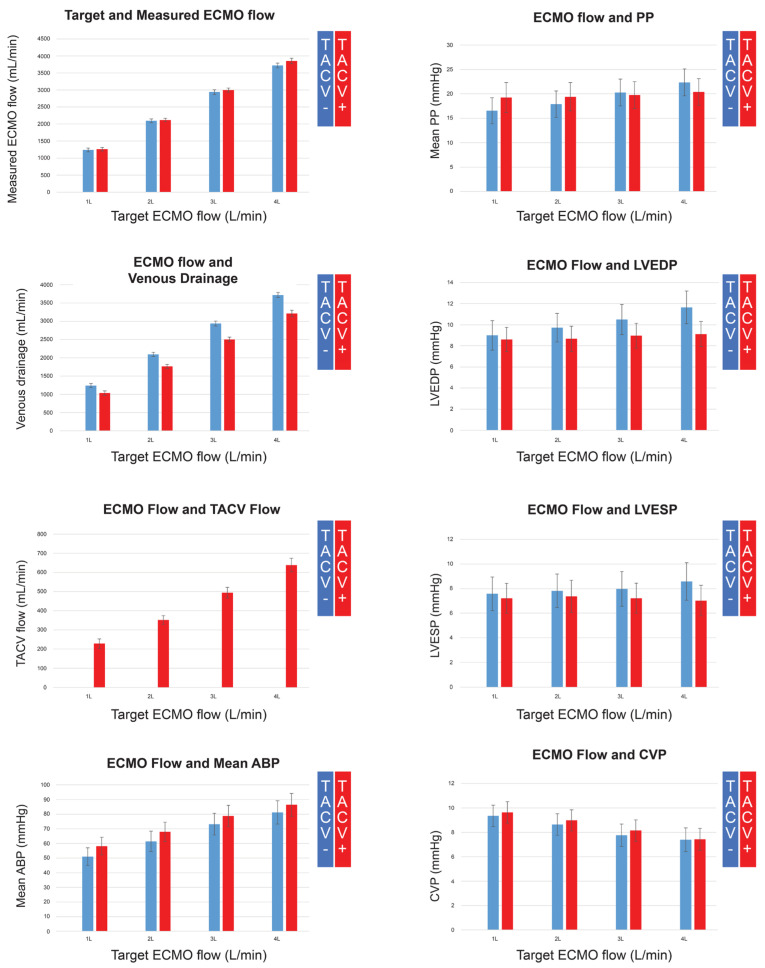
Extracorporeal membrane oxygenation (ECMO) flow, venous drainage, transaortic catheter venting (TACV) flow, mean arterial blood pressure (ABP), pulse pressure (PP), left ventricular end-diastolic pressure (LVEDP), left ventricular end-systolic pressure (LVESP), and central venous pressure (CVP), according to the changing ECMO flow without TACV (TACV−) or with TACV (TACV).

Under controlled numbers of hypoxic injury, volume challenge, norepinephrine, and labetalol infusion status, hemodynamic measurements were collected while ECMO flow was changed up and down from 1 L/min to 4 L/min by 1 L/min without (*n* = 80) or with TACV (*n* = 78) (Figure 2B). Changes in measurements compared with baseline when the ECMO flow was 1 L/min were analyzed (Table 4, Figure 4 and Figure A1).

While the measured ECMO flow was comparable for each target flow regardless of whether TACV was applied, the TACV flow increased depending on the ECMO flow, and venous drainage was as low as the TACV flow in cases with TACV. Although the increase in mean ABP according to the increase in ECMO flow did not differ between the two groups, pulse pressure was accentuated without TACV when the ECMO flow increased. During the increase in ECMO flow, LVEDP significantly increased without TACV compared to LVEDP with TACV.

When the change in LVEDP during ECMO flow adjustment was analyzed for correlations with other hemodynamic changes, the mean change in ABP showed the most significant positive correlation (Table 5). When the effects were analyzed using multivariate linear regression, the mean ABP, TACV application, initial CVP, and initial heart rate were significantly correlated with LVEDP change. ECMO flow itself did not show a significant correlation after adjusting for mean ABP changes.

## 4. Discussion

The primary result of our study was that the LVEDP decreased when TACV, accounting for 15% of the total ECMO flow, was applied during peripheral VA-ECMO. Although the mean LVEDP decrease by TACV was 1.2 mmHg, which was smaller compared to 5.4 mmHg by microaxial pump in the previous preclinical study, the decrease in LVEDP by TACV was maximized when baseline LVEDP was escalated [18]. Although systolic PAP was higher when TACV was applied, this increase could be attributed to the decreased systemic venous drainage owing to the Y-shape connection of the venous cannula and TACV without an additional pump head for TACV. The PAP was not significantly different in diastole, and the decreased LVEDP implies that the increased systolic PAP might not provoke pulmonary congestion. Furthermore, we focused on situations in which lowering the LVEDP was critical. In previous clinical studies on the prognosis of cardiogenic shock after myocardial infarction, an LVEDP > 20 mmHg was a significant prognostic factor for mortality [19]. Because our experimental model was a human-sized pig, effective LV venting in this situation is of interest. As Kitamura et al. [16]. suggested successful LV venting as 25–30% of the LV dimension, we set a decrease of more than 5 mmHg (25% of 20 mmHg) as effective LV venting, followed by further analysis.

As shown in Table 3, when the ECMO flow was higher, the pulse pressure was lower, the heart rate was lower before TACV, and the decrease in LVEDP by TACV was more prominent. Conversely, when ECMO flow was low, pulse pressure was high, heart rate was high, and the effect of TACV was suboptimal. Associated with a lower heart rate as a predictive factor for effective TACV, bradycardia may serve as a surrogate marker for more profound ischemic damage, particularly because hypoxic biventricular dysfunction was induced to create this cardiogenic shock model. Although some reports have advocated the prophylactic use of LV venting to prevent LV distension during ECMO support [20,21], our findings may provide cues for sparing this invasive procedure in selected patients. In contrast, an extreme drop in negative LV pressure isolated from aortic pressure and the rapid restoration of beating without cardiac compression in a standstill heart exemplify the critical role of direct LV venting in severe LV dysfunction [22] (Video S1). The idea that a venting strategy actually improves recovery is theoretically plausible but has not been clinically proven. Although the recovery of LV was not defined or measured in our experiment, the video above shows the prompt recovery of LV contraction following the application of TACV.

**Figure A2:**
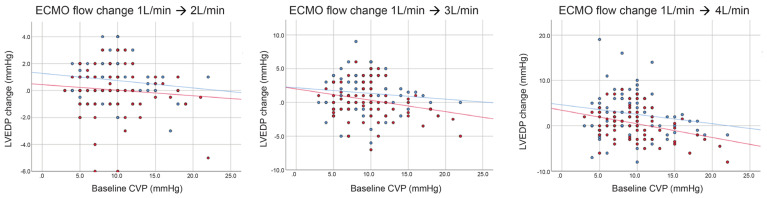
Change in left ventricular end-diastolic pressure (LVEDP) by extracorporeal membrane oxygenation (ECMO) flow with (red dots and lines) or without (blue dots and lines) transaortic catheter venting (TACV) according to baseline central venous pressure (CVP).

In our study, ECMO flow, mean ABP, and LVEDP were all directly proportional without TACV. However, with TACV, the LVEDP did not increase, whereas the mean ABP increased during ECMO flow escalation, and the LVESP showed a decreasing tendency. However, in a recent preclinical study by Hála et al. [23], excessive VA-ECMO flow negatively affected the LV workload. In their experimental model, the LV peak pressure was elevated by increasing ECMO flow, which cannot determine whether the systemic arterial pressure or flow itself has a negative effect on the LV. In our study, LVEDP change during ECMO flow change was analyzed using multivariate linear regression to determine whether this change was attributable to the flow itself or to increased mean ABP. In multivariate analysis, mean ABP was the most significant predictive factor, and heart rate, TACV application, and initial CVP also had an impact on LVEDP change. After adjusting for these relevant factors, ECMO flow itself did not act as an independent risk factor for LVEDP elevation. In some cases, LVEDP decreased while ECMO flow increased, even in the absence of TACV (blue dots under 0 in Figure A2). In these cases, the initial CVP was extremely high, and the LVEDP decreased more during ECMO flow escalation when the initial CVP was higher. ECMO flow escalation in the volume-overload status, but not afterload of the LV, and increased ECMO flow can relieve the LV in preload, even without venting.

VA-ECMO is an indispensable therapeutic option for cardiogenic shock. In acute and fulminant cardiogenic shock, peripheral VA-ECMO is an irreplaceable mechanical circulatory support that can be initiated without delay in the operating theater, catheterization laboratory, or even in the intensive care unit and at the emergency room bedside. However, retrograde flow counteracting the opening of the aortic valve and the inherent limitation of partial bypass, which cannot drain the left heart, cause great concern regarding LV overload in pressure and volume.

LV distension and an increased LVEDP can lead to clinical complications. First, a distended LV increases myocardial wall tension, which requires more myocardial workload, and myocardial perfusion pressure decreases owing to elevated LVEDP [1,2]. This results in prolonged subendocardial ischemia, which delays and even hinders recovery. Second, stagnation of blood flow in the LV promotes thrombosis in the LV, followed by massive thromboembolism [24,25,26]. Third, elevated LVEDP causes left-atrial hypertension and aggravates pulmonary edema [2]. Therefore, it is critical to prevent and manage LV distension in a timely manner, starting with non-invasive methods of volume management, ventilator settings, and inotropic/vasodilator usage. However, when LV dysfunction is severe, the aortic valve cannot open with medical optimization alone, and a more aggressive approach is needed to unload the LV.

Atrial septostomy and LA drainage with another venous cannula are widely performed for left-heart decompression. Although this method can relieve left-atrial hypertension and prevent pulmonary edema, Hasde et al. [27] showed that the decrease in pulmonary capillary wedge pressure was smaller in balloon atrial septostomy than in transapical LV vents in their clinical study. Atrial septostomy also requires patient transfer to the catheter laboratory and a highly specialized interventional cardiologist, while the early clinical experience of TACV showed the feasibility of TEE-guided bedside procedures. Atrial septostomy is an invasive procedure that can result in cardiac tamponade and unplanned surgery [28,29]. Additionally, the resolution of blood stasis in the LV and relief of LV distension are limited by this method.

**Figure 5:**
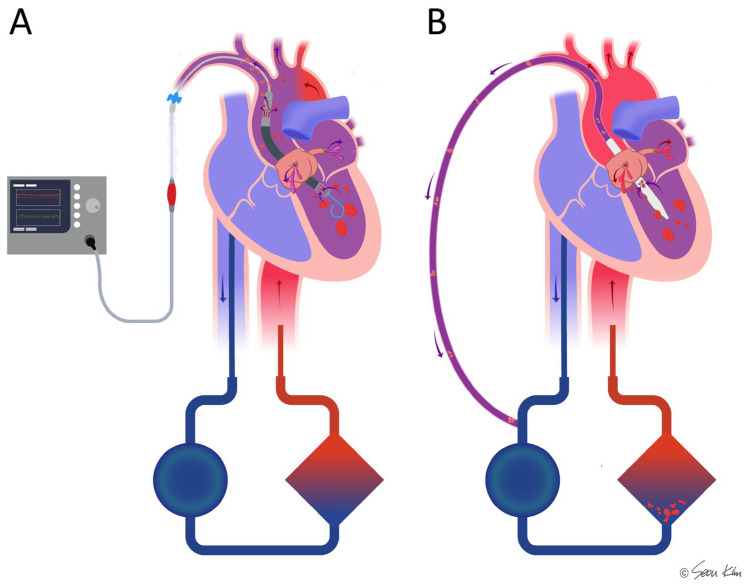
Comparison of the microaxial pump (Impella) and transaortic catheter venting (TACV) in combination with ECMO in cases where thrombi or hypoxemia are present in the left ventricle. In Impella-supported patients (**A**), there is a potential risk of hypoxic ischemia and thromboembolic complications due to direct ejection of the thrombi or desaturated blood. In contrast, TACV (**B**) may help mitigate these risks by facilitating drainage to the oxygenator and filter, thereby reducing the likelihood of embolic events and improving oxygenation.

Recently, direct LV venting using a catheter-delivered microaxial pump through the aortic valve (Impella) has been widely accepted as the most effective venting technique for peripheral VA-ECMO, known as ECMELLA or ECPELLA [10,30]. However, there are some situations when TACV has some benefits over Impella. When LV thrombus or hypoxemia due to severe pulmonary edema are combined, the microaxial pump has risks of acute brain injury [11,12] (Figure 5). Drainage into the oxygenator rather than ejection to the ascending aorta was safer for this patient to prevent embolism and differential hypoxia. In addition to this theoretical background, the high cost of single-use devices is a significant obstacle to implanting Impella for mechanical circulatory support in the real world, especially in developing countries. In South Korea, the use of this device has not been approved by the National Health Insurance Service owing to financial issues. For this reason, a few domestic clinical case series have attempted TACV as a method for direct LV venting. However, these case series compared the recovered static status of the LV after TACV rather than the direct effect of TACV on hemodynamic parameters [14,15]. Although earlier preclinical studies using small pigs were reported by Kitamura et al. [16], their experience was limited, and information about flow and LV pressure data was unavailable.

Considering the limitations of the above-mentioned studies, our study is unique in that the hemodynamic impact of TACV, including changes in LVEDP, was observed in various hemodynamic situations. All procedures and monitoring were conducted using SONO-guided percutaneous intervention without surgical incision [31]. Using this minimally invasive percutaneous approach, blood loss during the experiment was minimized, which enabled a long observation time and repetitive hemodynamic challenges, including multiple hypoxia inductions and preload/afterload control with volume challenge/vasoconstrictor adjustment. While alleviating the pain of each subject, multiple hemodynamic scenarios in a single subject could minimize the number of subjects required to induce meaningful results based on the consideration of animal rights in preclinical experiments.

Our study had several limitations. First, although we used the MVPS in the study design to include volume data, it could not be included in the analysis because the volume loading and change in ECMO flow affected the sensing of conductance, resulting in unreliable volume measurements. Second, transesophageal echocardiography was not performed in this experiment. MPVS and TACV catheters were both passed through the aortic valve, and aortic regurgitation might have had a hemodynamic effect. However, we could not evaluate regurgitation due to the unavailability of echocardiography. Third, the TACV catheter used in our experiment was custom-made and is not currently in clinical use because no effective commercial products are available for TACV. Alternatives include pigtail catheters for vascular intervention, whose internal diameter is too small to accommodate sufficient flow. Fourth, the approach for the LV was through the carotid artery in this study, which should be changed to the femoral artery in clinical applications owing to the risk of carotid artery cannulation associated with stroke, thromboembolism, and airway problems when bleeding. This was attributed to the unavailability of appropriate products in terms of length and size. As we elucidated the hemodynamic effects of TACV in this study, further research is necessary to develop a TACV catheter that could be long enough to be inserted through femoral access and large enough to draw 15% of the ECMO flow. In addition, the risk of thromboembolic complications associated with such indwelling catheters in the ascending aorta and aortic arch has to be addressed before their clinical application. Finally, the findings of this study should be interpreted with caution due to the small sample size, although we sought to mitigate this limitation by inducing repeated and multiple hemodynamic changes in each individual subject and accumulating a large number of observations for analysis.

## 5. Conclusions

TACV can drain up to 15% of the total ECMO flow and has a significant LV pressure unloading effect. When high ECMO flow is needed for end-organ perfusion, pulse pressure is diminished, and LVEDP is elevated, the effect of TACV is prominent. With TACV, higher ECMO flow and increased mean ABP do not overload the LV, enabling simultaneous LV recovery and end-organ perfusion. Because our model was a human-sized porcine model, the effects of TACV proven in this preclinical study are expected to be helpful in applying TACV and interpreting its effects in clinical settings.

## Figures and Tables

**Table 1:** Paired comparison of hemodynamic data between without transaortic catheter venting (TACV) and with TACV.

	Without TACV	With TACV	Δ with TACV–Without TACV (95% Confidence Interval)	*p*-Value
ECMO RPM (/min)	2312.2 ± 600.5	2307.8 ± 601.8	−4.2 (−7.2 to −1.3)	0.005
ECMO flow (mL/min)	2618.2 ± 985.1	2793.9 ± 1010.5	175.4 (161.2 to 189.6)	<0.001
TACV flow (mL/min)	-	434.1 ± 175.0	431.9 (417.4 to 446.4)	<0.001
Venous drain (mL/min)	2618.2 ± 985.1	2359.8 ± 892.4	−256.5 (−271.9 to −241.2)	<0.001
Systolic ABP (mmHg)	87.5 ± 36.8	86.0 ± 36.2	−1.5 (−2 to −1)	<0.001
Diastolic ABP (mmHg)	66.9 ± 28.8	67.3 ± 28.5	0.4 (0 to 0.7)	0.032
Mean ABP (mmHg)	74.0 ± 31.8	73.6 ± 31.3	−0.3 (−0.7 to 0)	0.058
Pulse pressure (mmHg)	20.5 ± 12.8	18.7 ± 12.8	−1.8 (−2.1 to −1.5)	<0.001
Systolic PAP (mmHg)	33.2 ± 12.3	34.1 ± 12.4	0.9 (0.5 to 1.2)	<0.001
Diastolic PAP (mmHg)	13.6 ± 9.6	13.8 ± 9.8	0.2 (0 to 0.4)	0.066
Mean PAP (mmHg)	22.3 ± 9.0	22.8 ± 9.1	0.5 (0.3 to 0.8)	<0.001
CVP (mmHg)	9.3 ± 4.1	9.3 ± 4.0	0 (−0.1 to 0.1)	0.593
LVEDP (mmHg)	8.7 ± 6.0	7.6 ± 5.4	−1.2 (−1.4 to −1)	<0.001
LVESP (mmHg)	6.5 ± 5.8	5.4 ± 5.7	−1.1 (−1.3 to −0.9)	<0.001
HR (/min)	87.9 ± 20.5	88.7 ± 21.3	0.3 (−0.5 to 1.2)	0.465

Continuous variables were summarized as mean ± standard deviation. ECMO, extracorporeal membrane oxygenation; ABP, arterial blood pressure; PAP, pulmonary artery pressure; CVP, central venous pressure; LVEDP, left ventricular end-diastolic pressure; LVESP, left ventricular end-systolic pressure; HR, heart rate.

**Table 2:** Pearson correlation coefficient and results of multivariate linear regression analysis for change of left ventricle end-diastolic pressure (ΔLVEDP) by transaortic catheter venting (TACV).

	Pearson Coefficient “R”	*p*-Value	Beta (95% Confidence Interval)	*p*-Value
ECMO flow (mL/min)	−0.135	<0.001	−4.51 × 10^−4^ (−0.001 to 0)	<0.001
TACV flow (mL/min)	−0.259	<0.001		
Systolic ABP (mmHg)	−0.09	0.028		
Diastolic ABP (mmHg)	−0.086	0.037		
Mean ABP (mmHg)	−0.088	0.033	0.012 (0.006 to 0.019)	<0.001
Pulse pressure (mmHg)	−0.067	0.105		
Systolic PAP (mmHg)	0.042	0.31		
Diastolic PAP (mmHg)	−0.027	0.516		
Mean PAP (mmHg)	0.013	0.758		
CVP (mmHg)	−0.093	0.023	0.116 (0.065 to 0.168)	<0.001
LVEDP (mmHg)	−0.437	<0.001	−0.256 (−0.297 to −0.214)	<0.001
LVESP (mmHg)	−0.363	<0.001		
HR (/min)	0.220	<0.001	0.008 (−0.001 to 0.018)	0.098

ECMO, extracorporeal membrane oxygenation; ABP, arterial blood pressure; PAP, pulmonary artery pressure; CVP, central venous pressure; LVEDP, left ventricular end-diastolic pressure; LVESP, left ventricular end-systolic pressure; HR, heart rate.

**Table 3:** Comparison of baseline hemodynamic data between ineffective (ΔLVEDP > −5 mmHg) and effective (ΔLVEDP ≤ −5 mmHg) transaortic catheter venting (TACV) in a high baseline LVEDP (>20 mmHg) setting.

	Ineffective TACV (*n* = 16)	Effective TACV (*n* = 6)	*p*-Value
ECMO flow (mL/min)	2550 (1750–3150)	3700 (3400–3900)	0.046
Systolic ABP (mmHg)	138 (128–150.5)	124.5 (81–159)	0.555
Diastolic ABP (mmHg)	94 (88–108.5)	104.5 (72–121)	0.712
Mean ABP (mmHg)	112.5 (108.5–123.5)	110.5 (76–132)	0.825
Pulse pressure (mmHg)	46.5 (36.5–48)	25 (17–31)	0.022
Systolic PAP (mmHg)	34 (32–38.5)	31.5 (23–44)	0.284
Diastolic PAP (mmHg)	15 (12.5–17)	15.5 (10–23)	0.941
Mean PAP (mmHg)	23 (22.5–26)	23.5 (16–32)	0.629
CVP (mmHg)	14 (10.5–16)	11.5 (9–15)	0.46
LVEDP (mmHg)	22 (21.8–23.5)	28 (26–38)	0.001
LVESP (mmHg)	19.5 (17.5–20.8)	26 (20–35)	0.046
HR (/min)	91.5 (88–99)	73 (72–77)	0.002

Continuous variables were described as the median and interquartile range (25th to 75th percentile), and their differences were tested using the Mann–Whitney U test due to non-normal distribution. ECMO, extracorporeal membrane oxygenation; ABP, arterial blood pressure; PAP, pulmonary artery pressure; CVP, central venous pressure; LVEDP, left ventricular end-diastolic pressure; LVESP, left ventricular end-systolic pressure; HR, heart rate.

**Table 4:** Difference in change in hemodynamic measurements during an increase in extracorporeal membrane oxygenation flow with and without transaortic catheter venting.

		Without TACV	With TACV	*p*-Value
ECMO Flow Control		(*n* = 78)	(*n* = 80)	
1 L/min → 2 L/min	ECMO RPM change (/min)	558.7 ± 122.1	525.9 ± 130.1	0.104
	ECMO flow change (mL/min)	859 ± 208.5	852.5 ± 177.2	0.834
	TACV flow change (mL/min)	0 ± 0	118.9 ± 93.8	<0.001
	Venous flow change (mL/min)	859 ± 208.5	733.6 ± 179.2	<0.001
	Systolic ABP change (mmHg)	11.3 ± 14.2	9.6 ± 13.1	0.432
	Diastolic ABP change (mmHg)	9.9 ± 12.2	9.6 ± 11.2	0.879
	Mean ABP change (mmHg)	10.4 ± 12.7	9.6 ± 11.7	0.672
	Pulse pressure change (mmHg)	1.4 ± 4	0 ± 4.1	0.029
	Systolic PAP change (mmHg)	0.8 ± 4.3	1 ± 6.5	0.782
	Diastolic PAP change (mmHg)	−0.6 ± 1.7	0 ± 5.5	0.344
	Mean PAP change (mmHg)	0 ± 2.6	−0.5 ± 6.4	0.578
	CVP change (mmHg)	−0.7 ± 0.8	−0.7 ± 0.7	0.967
	LVEDP change (mmHg)	0.8 ± 1.5	0 ± 1.8	0.006
	LVESP change (mmHg)	0.3 ± 1.6	0.1 ± 2.6	0.692
	HR change (/min)	0.8 ± 8.1	0.1 ± 9.8	0.623
1 L/min → 3 L/min	ECMO RPM change (/min)	1103.6 ± 187.3	1043 ± 158.2	0.029
	ECMO flow change (mL/min)	1700.6 ± 295.7	1736.3 ± 280.3	0.438
	TACV flow change (mL/min)	0 ± 0	259.5 ± 110	<0.001
	Venous flow change (mL/min)	1700.6 ± 295.7	1476.7 ± 263.9	<0.001
	Systolic ABP change (mmHg)	24.7 ± 20.4	21 ± 20.7	0.254
	Diastolic ABP change (mmHg)	20.9 ± 17.2	20.6 ± 17.6	0.904
	Mean ABP change (mmHg)	22.2 ± 18.4	20.6 ± 18.9	0.595
	Pulse pressure change (mmHg)	3.8 ± 6.9	0.4 ± 5.9	0.001
	Systolic PAP change (mmHg)	1.6 ± 7.7	1.7 ± 7.4	0.952
	Diastolic PAP change (mmHg)	−1.4 ± 4.8	−0.6 ± 6.7	0.362
	Mean PAP change (mmHg)	0.1 ± 5.6	0 ± 5.7	0.954
	CVP change (mmHg)	−1.6 ± 1.3	−1.5 ± 1	0.780
	LVEDP change (mmHg)	1.4 ± 2.9	0.4 ± 2.6	0.023
	LVESP change (mmHg)	0.3 ± 2.9	0 ± 2.4	0.475
	HR change (/min)	1.6 ± 15	1.8 ± 13.5	0.904
1 L/min → 4 L/min	ECMO RPM change (/min)	1626.4 ± 203.4	1606.5 ± 230.4	0.567
	ECMO flow change (mL/min)	2482.1 ± 322.6	2588.8 ± 353.6	0.049
	TACV flow change (mL/min)	0 ± 0	402.1 ± 138.6	<0.001
	Venous flow change (mL/min)	2482.1 ± 322.6	2186.6 ± 331	<0.001
	Systolic ABP change (mmHg)	34.2 ± 26.4	28.8 ± 27.2	0.214
	Diastolic ABP change (mmHg)	28.3 ± 22.1	27.8 ± 23.7	0.884
	Mean ABP change (mmHg)	30.2 ± 23.7	28.1 ± 25.1	0.597
	Pulse pressure change (mmHg)	5.9 ± 8.7	1.1 ± 7.9	<0.001
	Systolic PAP change (mmHg)	0.5 ± 8.8	0.4 ± 8.4	0.934
	Diastolic PAP change (mmHg)	−1.6 ± 5.1	−1.4 ± 5.7	0.849
	Mean PAP change (mmHg)	−0.8 ± 5.4	−1 ± 5.4	0.857
	CVP change (mmHg)	−1.9 ± 1.7	−2.2 ± 1.6	0.227
	LVEDP change (mmHg)	2.7 ± 4.9	0.5 ± 3.4	0.002
	LVESP change (mmHg)	1 ± 4.1	−0.2 ± 3.3	0.042
	HR change (/min)	0.4 ± 14.4	−0.2 ± 13.8	0.794

Continuous variables were summarized as mean ± standard deviation. RPM, revolutions per minute; ABP, arterial blood pressure; PAP, pulmonary artery pressure; CVP, central venous pressure; LVEDP, left ventricle end-diastolic pressure; LVESP, left ventricle end-systolic pressure; HR, heart rate; ECMO, extracorporeal membrane oxygenation; TACV, transaortic catheter venting.

**Table 5:** Pearson correlation coefficient and results of multivariate linear regression analysis for change in left ventricle end-diastolic pressure compared to the value when the extracorporeal membrane oxygenation flow was 1 L/min.

	Pearson Coefficient R	*p*-Value	Beta (95% CI)	*p*-Value
ECMO flow change (mL/min)	0.123	0.007	0.000 (−0.001 to 0)	0.191
Mean ABP change (mmHg)	0.423	<0.001	0.076 (0.063 to 0.089)	<0.001
With TACV *	−0.204	<0.001	−0.954 (−1.424 to −0.484)	<0.001
Initial CVP (mmHg)	−0.185	<0.001	−0.115 (−0.202 to −0.027)	0.010
Initial LVEDP (mmHg)	−0.182	<0.001	0.034 (−0.028 to 0.097)	0.277
Initial HR (mmHg)	−0.274	<0.001	−0.051 (−0.062 to −0.041)	<0.001

ECMO, extracorporeal membrane oxygenation; TACV, transaortic catheter venting; ABP, arterial blood pressure; PAP, pulmonary artery pressure; CVP, central venous pressure; LVEDP, left ventricle end-diastolic pressure; LVESP, left ventricle end-systolic pressure; HR, heart rate. * Compared to without.

## Data Availability

The original data supporting the findings of this study are openly available at the following URLs: https://1drv.ms/x/s!AhU7YNE0HdUqgpZ9nvkJCK1qiky8Jw (accessed on 19 March 2019); https://1drv.ms/x/s!AhU7YNE0HdUqgpcSfA-OVUAUpmQz4A (accessed on 19 March 2019).

## Supplementary Materials

The following supporting information can be downloaded at https://www.mdpi.com/article/10.3390/medicina61040552/s1, Video S1. Extreme changes in left ventricular end-diastolic pressure (LVEDP) by transaortic catheter venting (TACV) when extracorporeal membrane oxygenation (ECMO) flow and mean arterial blood pressure were high and pulse pressure was low. LVESP, left ventricular end-systolic pressure; LV, left ventricle.

## Appendix A

**Table A1:** Baseline characteristics of the 11 pigs analyzed.

Characteristics	Median (Interquartile Range)
Body weight (kg)	55 (51–65)
Initial systolic ABP (mmHg)	109 (96–117)
Initial diastolic ABP (mmHg)	74 (61–77)
Initial mean ABP (mmHg)	87 (74–91)
Initial systolic PAP (mmHg)	24 (21–29)
Initial diastolic PAP (mmHg)	17 (9–19)
Initial mean PAP (mmHg)	22 (17–23)
Initial CVP (mmHg)	7 (5–9)
Initial LVEDP (mmHg)	10 (6–10)
Initial LVESP (mmHg)	2 (0–6)
Initial HR (/min)	89 (85–93)
Initial cardiac output (L/min)	4.6 (3.7–5.1)
Body temperature (°C)	36.2 (36.0–36.8)
Initial pH	7.5 (7.5–7.6)
Initial pCO2 (mmHg)	32.6 (24.9–37)
Initial O2 (mmHg)	325.4 (275–348.5)
Initial Hematocrit (%)	30 (29–34)
Initial Lactate (mmol/L)	1.6 (1.2–2.2)
Initial Glucose (mg/dL)	101 (64–123)
Initial Na+ (mmol/L)	138.2 (137.3–138.4)
Initial K+ (mmol/L)	3.8 (3.4–4.1)
Initial iCa2+(mmol/L)	1.2 (1.1–1.4)
Time until ECMO run (min)	75.3 (68.7–79.9)
Time until TACV (min)	114.9 (77.9–148.1)
Experiment time (min)	392.5 (314.1–457.1)
Observed events	150 (95–222)

ABP, arterial blood pressure; PAP, pulmonary artery pressure; CVP, central venous pressure; LVEDP, left ventricular end-diastolic pressure; LVESP, left ventricular end-systolic pressure; HR, heart rate; ECMO, extracorporeal membrane oxygenation; TACV, transaortic catheter venting.
